# Harnessing adenovirus in cancer immunotherapy: evoking cellular immunity and targeting delivery in cell-specific manner

**DOI:** 10.1186/s40364-024-00581-1

**Published:** 2024-03-25

**Authors:** Miao Zeng, Wei Zhang, Yisheng Li, Li Yu

**Affiliations:** 1 Department of Hematology and Oncology, Shenzhen University General Hospital, International Cancer Center, Hematology Institution of Shenzhen University, Shenzhen University Medical School, Shenzhen University, Shenzhen, Guangdong 518000 China; 2 Guangdong Key Laboratory for Biomedical Measurements and Ultrasound Imaging, School of Biomedical Engineering, Shenzhen University Health Science Center, Shenzhen, 518060 China; 3 Shenzhen Haoshi Biotechnology Co., Ltd. No, 155 Hongtian Road, Xinqiao Street, Bao’an District, Shenzhen, Guangdong 518125 China

**Keywords:** Recombinant adenovirus, Oncolytic adenovirus, Adoptive cell therapy, Targeted-delivery, Tumor immunogenicity, Tumor microenvironment

## Abstract

Recombinant adenovirus (rAd) regimens, including replication-competent oncolytic adenovirus (OAV) and replication-deficient adenovirus, have been identified as potential cancer therapeutics. OAV presents advantages such as selective replication, oncolytic efficacy, and tumor microenvironment (TME) remodeling. In this perspective, the principles and advancements in developing OAV toolkits are reviewed. The burgeoning rAd may dictate efficacy of conventional cancer therapies as well as cancer immunotherapies, including cancer vaccines, synergy with adoptive cell therapy (ACT), and TME reshaping. Concurrently, we explored the potential of rAd hitchhiking to adoptive immune cells or stem cells, highlighting how this approach facilitates synergistic interactions between rAd and cellular therapeutics at tumor sites. Results from preclinical and clinical trials in which immune and stem cells were infected with rAd have been used to address significant oncological challenges, such as postsurgical residual tumor tissue and metastatic tissue. Briefly, rAd can eradicate tumors through various mechanisms, resulting from tumor immunogenicity, reprogramming of the TME, enhancement of cellular immunity, and effective tumor targeting. In this context, we argue that rAd holds immense potential for enhancing cellular immunity and synergistically improving antitumor effects in combination with novel cancer immunotherapies.

## Introduction

Recombinant adenovirus (rAd) represents an attractive candidate for virus vaccination and cancer immunotherapy. For viral vaccination, rAd can act as a vehicle to deliver specific viral antigens into host cells, prompting the immune system to recognize and build defenses against future infections. For example, rAd-based vaccine for COVID-19, which utilizes the SARS-CoV-2 spike protein as an antigen, has demonstrated specific antibody and T-cell responses in clinical trials [1, 2]. In the realm of cancer immunotherapy, given the nuanced differences between cancer cells and their normal counterparts, the challenges are distinct. The antigens delivered by rAd usually encompass tumor-associated antigens (TAAs) or tumor-specific antigens (TSAs) such as cancer-testis antigen NY-ESO-1 and melanoma-associated cancer-testis antigen (MAGE-A3) (NCT04908111) [3], as well as personalized patient-specific or shared neoantigens (ClinicalTrials.gov: NCT03639714, NCT03953235) [4]. In another aspect, the conditionally replicating adenovirus (CRAd), clinically referred to as oncolytic adenovirus (OAV), is engineered to specifically target and annihilate tumor cells for destruction, preventing their growth and spread. Furthermore, OAV also plays a role in reshaping the tumor microenvironment (TME) and bolstering cellular immunity [5–7]. By harnessing the complete potential of OAV in tumors, different strategies have been used to leverage their entry, replication, and lysis capabilities to maximize antitumor immunity [8–11].

Clinically, there are basically two primary methods for rAd administration, intravenous and localized injection. Although intravenous administration of rAd is encumbered by numerous challenges, such as neutralizing antibodies (NAbs), cytokine storm syndrome, disseminated intravascular coagulation, thrombocytopenia, and hepatotoxicity, direct intravenous administration of a high titer of rAd was superior to other routes for transgene expression [12, 13]. The existing methods for delivering viruses, such as nanoparticles and PEG/lipids/calcium phosphate, have been demonstrated to allow a high dose of viruses to be administered intravenously and increase therapeutic efficacy without inducing toxicity [14, 15]. In other cases, studies of localized injection, especially intraperitoneal administration of OAV indicated benefits in treating peritoneal metastasis over systemic injections, given its ability to deliver high concentrations directly to the tumor [16–18]. As such, the primary focus in potentiating rAd immunotherapy lies in balancing efficacy with safety, leading to tailored modifications in the viral genome to boost cellular immunity.

Adoptive cell therapy (ACT) is a type of vibrant tumor immunotherapy that involves a broad spectrum of immune cells, including dendritic cells (DCs), chimeric antigen receptor macrophages (CAR-Ms), T-cell receptor-engineered T cells (TCR-T cells), chimeric antigen receptor T cells (CAR-T cells), cytokine-induced killer cells (CIKs), cytotoxic T lymphocytes (CTLs), tumor-infiltrating lymphocytes (TILs), and natural killer cells (NKs) [19]. Both rAd and retrovirus serve as prevailing vectors in ACT, with their selection hinging on distinct treatment objectives and disease profiles. Retrovirus, which integrates into the host genome, enables prolonged expression. Conversely, rAd can be applied *in vivo* with low toxicity, transient expression and effective infection. Significantly, working in combination with ACT, rAd can synergistically stimulate immunogenicity in the TME to increase ACT efficacy [20–22], or reach tumors through infused cells to cause oncolysis through a phenomenon known as “viral hitchhiking”, by loading on the cell surface [23] or infecting cells [24, 25]. In this regard, stem cells with excellent tumor-homing properties usually serve as a promising systemic delivery tool for rAd and demonstrate safety and efficacy against tumors [26–30].

To potentiate antitumor immunity, it is vital to elicit tumor immunogenicity, synergize with cellular therapies, modulate the TME and target tumor sites. This review focuses on the generating and strengthening of rAd immunotherapies and provides a comprehensive understanding of its anti-tumor distinctions and mechanisms. Notably, we highlight promising strategies in combination with ACT to improve immunotherapeutic efficacy, which are expected to provide promising approaches for successful cancer treatment.

## Biological characteristics of adenovirus and the development of rAd vectors

**Fig. 1:**
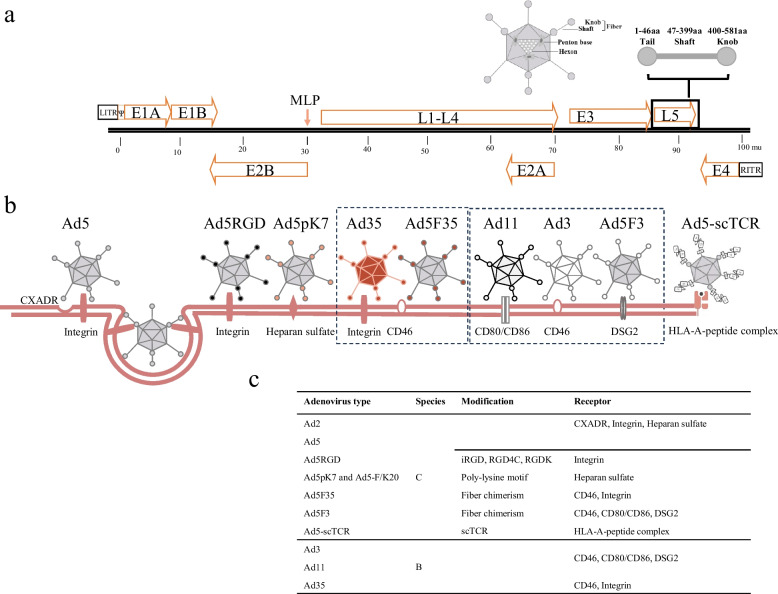
The genome structure and entry receptors of adenovirus. **a** Schematic representation of Ad5 genome and the major capsid proteins. **b** Cellular uptake and transduction mediators of various types of adenoviral vectors. The Ad5 primarily infects cells via both CXADR and integrin. The Ad5 can be engineered to increase cell entry by incorporating peptide into capsid and swapping fibers of other species of adenovirus. Surface receptors for cell binding of B, D subgroup and chimeric adenoviruses are displayed. **d** Summary of adenovirus entry receptors and fiber modifications.

Adenovirus is a nonenveloped DNA virus with a 36 kb genome that contains early genes (E1-E4, pIX and pIVa2) associated with viral replication and five late genes (L1-L5) involved in assembly. To date, 7 subgroups (subgroups A-G) and over 100 human adenovirus genotypes have been identified (http://hadvwg.gmu.edu/). The adenoviral capsid features a regular 20-sided structure comprising hexons, pentons, and fibers, as well as other small proteins such as pIX, pIIIa and pIVa2 (Fig. 1a). Human adenovirus type 5 (Ad5, subgroup C) infection primarily depends on the interaction between coxsackievirus-adenovirus receptor (CXADR) on the cell surface and fibers on the viral capsid protein. Ad5 endocytosis into target cells occurs through two steps: the binding of a fiber knob to CXADR, and the subsequent interaction between the Arg-Gly-Asp motif (RGD) located in the penton base and integrin subunits alpha V (αv), beta 3 (β3) and beta 5 (β5) (Fig. 1b) [31]. Other receptors, such as CD80/CD86, CD46, desmoglein 2 (DSG2) and sialic acid, are involved in infection by B or D subgroup adenoviruses (Fig. 1b) [32].

rAd has been continuously modified to enhance gene capacity, infection efficiency, duration of gene transduction and safety. The first-generation rAd lacks E1 and E3 regions and thus is replication incompetent and innate-immunity attenuated. The Ad5 vector from the first generation is predominantly used in scientific research and clinic [33, 34]. To mitigate the robust immune response, the second-generation rAd sees further gene deletions and an expanded transgenic space. However, deletion of E2 and/or E4 genes can lead to a decreased viral titer [34]. The third-generation rAd only retains the inverted terminal repeats (ITR) and packaging signal sequences, which can accommodate up to 37kb of exogenous DNA with the aid of a helper-packaging virus [35, 36]. This high-capacity rAd minimizes residual gene expression, attenuates host immune, and achieves sustained *in vivo* transgenic expression. Its vast loading capability also allows it to carry prominent gene-editing systems like CRISPR/Cas9 and TALENs.

## Construction and Modification Strategies for OAV

OAV is engineered to possess capabilities of direct oncolysis and immune induction. Briefly, they can cause immunogenic cell death (ICD) and trigger the production of proinflammatory cytokines, pathogen-associated molecular patterns (PAMPs), and damage-associated molecular patterns (DAMPs) from dying cancer cells, which can activate DCs and T-cells to destruct tumor. Improving the targeting and replication of OAV is an effective approach to enhance its oncolytic effects.

### Entry and targeting modifications

#### Capsid peptide incorporation

The CXADR required for Ad5 entry is typically expressed at low levels in target cells, prompting researchers to explore alternative entry pathways [37–39]. The HI loop or COOH-terminus of the fiber knob, and the hypervariable region of the hexon and shaft are promising candidates for displaying foreign peptide sequences [40, 41]. The RGD motif and its extended versions, including the internalized RGD (iRGD, CRGDKGPDC), RGD4C (CDCRGDCFC) and RGDK, have been used extensively to favor adenovirus entry via the integrin-mediated pathway (Fig. 1b) [42–44]. Some cell-permeable peptides, such as the polylysine motif (pK7) and a stretch of 20 lysine residues (F/K20) incorporated into the fiber have also shown an improved transduction efficiency (Fig. 1b) [45–48].

#### Chimeric rAd engineering

Chimeric rAd can alter viral tropism and is a potent instrument for devising immune cell-based vaccination approaches. Table 1 lists the chimeric rAd in preclinical and clinical trials. The directed-evolution screened Enadenotucirev (formerly ColoAd1) comprises the Ad11 capsid structure from subgroup B and a chimeric E2B from Ad11/Ad3, which allows it to avoid the Ad5 NAbs and display robust tumor cell eradication via intravenous administration [49–55]. In the case of OAV ONCOS-102, integrates the Ad3 fiber knob domain into the Ad5 structure (Ad5F3). Therefore, Ad5F3 derives superior tropism via the adequately expressed Ad3 receptor (Fig. 1b) [9, 56, 57]. Derived from Ad5, Ad5F35 replaces its receptor binding site with the fiber of Ad35 and strongly increases entry into immune cells by shifting its cellular receptor from CXADR to the ubiquitously expressed complement receptor CD46 (Fig. 1b). Lokon oncolytic adenovirus (LOAd) is Ad5F35 and shows promise in cancer intervention [6, 58–62].

**Table 1:** Preclinical and clinical trials using chimeric rAd.

**Cancer**	**Vector**	**Construct**	**Transgene/Features**	**Administration**	**Phase**	**ClinicalTrials.gov ID**	**Ref.**	**Combination**
Ovarian cancer	Enadenotucirev (ColoAd1)	Ad11p/Ad3		Intravenous	Phase I	NCT02028117	[50]	Paclitaxel
Colorectal cancer Head and neck cancer Epithelial tumor				Intravenous	Phase I	NCT02636036	[51]	Nivolumab
Rectal cancer				Intravenous	Phase I	NCT03916510	[52]	Capecitabine, radiotherapy
Lung cancer		NG-347 Ad11p/Ad3	IFNα, MIP1α and CD80		Preclinical		[53]	CAR-T
Epithelial tumor		NG-348 Ad11p/Ad3	Anti-CD3 and anti-CD80	Intravenous	Phase II	NCT02028442	[49]	
Colon carcinoma Lung cancer Bladder cancer		NG-641 Ad11p/Ad3	CD40L, FAP/CD3, CXCL9/10, IFNα	Intravenous, Intratumoral	Phase I	NCT02053220	[54]	Tumor resection
Metastatic cancer Epithelial tumor		NG-350A	CD40L	Intravenous, Intratumoral	Phase I	NCT03852511	[55]	
Ovarian cancer	ONCOS-102	Ad5/3-∆24-GM-CSF	GM-CSF	Intracavitary	Preclinical		[9]	
Peritoneal malignancy				Intraperitoneal	Phase II	NCT02963831		Durvalumab, cyclophosphamide
Solid tumor				Intratumoral	Phase I	NCT01598129	[63]	
Mesothelioma				Intratumoral	Phase II	NCT02879669	[64]	Pemetrexed, ​cisplatin
Multiple myeloma	LOAd700	Ad5/35-E2F-∆24-CD40L	CD40L		Preclinical		[58]	
Solid tumor	LOAd703	Ad5/35-E2F-∆24-CD40L-4-1BBL	CD40L, 4-1BBL	Intratumoral	Phase II Phase II Phase II	NCT03225989 NCT04123470 NCT03555149		Atezolizumab, regorafenib, imprime PGG
Pancreatic cancer					Phase II	NCT02705196		Gemcitabine, nab-paclitaxel
Pancreatic cancer				Peritumoral	Preclinical		[61, 62]	Gemcitabine
Lymphoma				Intratumoral	Preclinical		[59]	CAR-T
Melanoma	Exosome-LOAd703	Ad5/35-E2F-∆24-CD40L-4-1BBL	Exosome delivery	Intratumoral	Preclinical		[60]	
Melanoma	LOAd732	Ad5/35-E2F-∆24-CD40L-4-1BBL-IL-2	CD40L, 4-1BBL, IL-2	Intratumoral	Preclinical		[6]	

#### Tumor-targeted OAV modification

Another strategy for engineering tumor-targeted OAV involves incorporating a single-chain T-cell receptor (scTCR) specific for TAA into adenoviral fibers. This approach allowed CXADR-independent viral entry and selectively infected cells that presented the corresponding TAA within the context of human leukocyte antigen (HLA)-A and effectively eradicated tumors [65].

### Conditional replication modifications

**Fig. 2:**
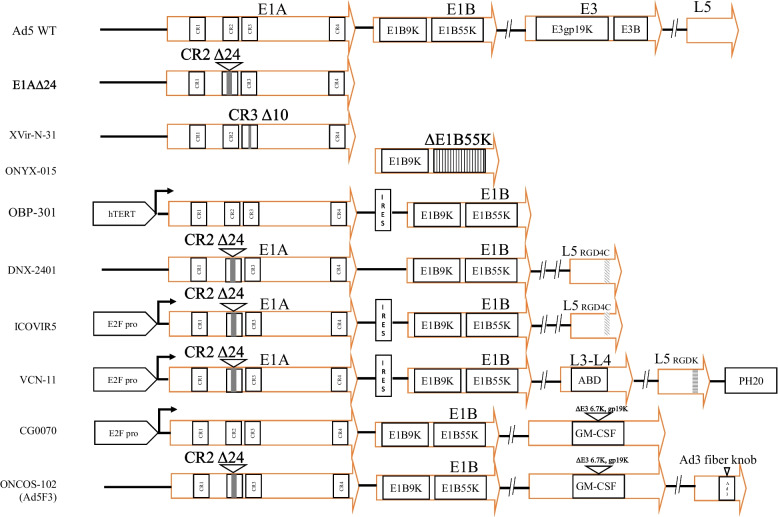
Engineered OAV-based gene delivery platform. Representatives of OAV modifications are depicted. Strategies of OAV replication and entry include the E1A∆24, ΔE1A 13S CR3, ΔE1B55K and specific promoter regulation of E1A as well as capsid modification

Adenovirus E1 is composed of E1A and E1B and initiates viral replication through interactions with cellular proteins. OAV that can conditionally activate E1 genes for replication within tumor cells is designed to selectively proliferate within and destroy tumor cells. There are generally four strategies for OAV to achieve conditional replication: a) a 24 bp constant deletion in the E1A conserved region 2 (E1A∆24), b) a conserved region 3 (CR3) deletion of the transactivator protein E1A 13S, c) an E1B55K deletion or E1B-93R mutation, d) specific promoter regulation of E1A (Fig. 2).

#### E1A gene modification

E1A proteins are among the first to be expressed after adenoviral infection for initiation of the viral life cycle, cell cycle modulation, and transactivation of viral and cellular genes. Normally, adenoviruses can infect and propagate in dormant cells in which the E1A CR2 interacts with the retinoblastoma protein (Rb), releasing the E2F transcription factor and advancing the cell from G1 to S-phase [66–68]. The strategic E1A∆24 hampers its ability to engage with functional Rb, rendering the adenovirus incapable of replicating in normal cells. However, tumor cells, characterized by excessive E2F due to the dysregulation of antioncogene Rb, support Ad5-∆24 replication [69, 70]. Both intratumoral and intravenous administration of Ad5-∆24 proved to be relatively safe and somewhat effective when injected into solid tumors [71, 72]. Table 2 summarizes the canonical OAV with E1A∆24.

**Table 2:** OAVs with a 24 bp deletion in E1A

**Cancer**	**Vector**	**Construct**	**Transgene/Feature**	**Administration**	**Phase**	**ClinicalTrials.gov ID**	**Ref.**	**Combination**
Solid tumor	CAdVEC	Ad5-∆24		Intratumoral	Phase I	NCT03740256	[71]	HER2-CAR-T
Breast cancer				Intravenous	Preclinical		[72]	
Solid tumor		Ad5-∆24-GM-CSF	GM-CSF	Intratumoral, intracavitary	Preclinical		[73]	
Glioma	DNX-2401	Ad5-∆24-RGD4C		Intratumoral, intramural	Phase I	NCT03178032 NCT01956734	[74, 75]	Radiotherapy, chemotherapy
Glioblastoma				Intratumoral	Phase II	NCT02798406	[76]	Pembrolizumab
Glioma	DNX-2440	Ad5-Δ24-RGD4C-OX40L	OX40L	Intratumoral	Phase I	NCT03714334		
Colorectal liver metastasis				Intratumoral	Phase I	NCT04714983		
Lung cancer		Exosome-Ad5-∆24-CpG	Exosome delivery, CpG	Intravenous	Preclinical		[77, 78]	Paclitaxel

OAV with CR3 deletion in E1A 13S exhibited high replication potential in Y-box binding protein 1 (YB-1)-positive cancer cells but replication defects in normal cells. Its utility is exemplified by XVir-N-31 and its derivatives for glioma and bladder cancer (Table 3) [79–85]. Mechanistically, YB-1 is a multifunctional protein involved in a multitude of cellular processes, including transcriptional regulation, mRNA splicing, and translation. The involvement of YB-1 in various stages of mRNA metabolism makes it an attractive collaborator for viruses, which often hijack the host cellular machinery for replication and propagation [86, 87].

**Table 3:** OAVs with E1A 13S, E1B19K or E1B55K deletion

Cancer	**Vector**	**Construct**	**Transgene/Features**	**Administration**	**Phase**	**ClinicalTrials.** **gov ID**	**Ref.**	**Combination**
Bladder cancer	XVir-N-31	Ad5-Delo3-RGD	∆E1A13S, ∆E1B19K	Intratumoral	Preclinical		[82]	Ultrasound guidance
Bladder cancer Murine ewing sarcoma				Intratumoral	Preclinical		[83]	CDK4/6 inhibitor
Glioma				Intratumoral	Preclinical		[81]	Temozolomide
Glioblastoma	XVir-N-31-anti-PD-L1	Ad5-Delo3-RGD-PD-L1	∆E1A13S, ∆E1B19K, PD-L1 anbibody	Intratumoral	Preclinical		[85]	Nivolumab
Malignant ascites	Oncorine (H101)		∆E1B55K, partial E3 deletion	Intraperitoneal	Phase II	NCT04771676	[88]	
Bladder cancer				Intravesical	Phase II	NCT05564897		Camrelizumab
Liver cancer				Transarterial	Phase III	NCT03780049	[89]	Chemoembolization
Liver cancer				Transarterial	Phase I	NCT05675462		Tislelizumab, lenvatinib
Colorectal cancer				Intratumoral	Preclinical		[90]	Anti-PD-1 monotherapy
Colorectal cancer	ONYX-015 (dl520)		Ad2/Ad5, ∆E1B55K	Intravenous	Preclinical		[91]	5-FU/leucovorin
Garcinoma metastatic to liver				Intraarterial	Phase II		[92]	
Cervical cancer				Intratumoral	Preclinical	ChiCTR-OPC-15006142	[13]	Chemoradiotherapy
Carcinoma metastatic to lung				Intravenous	Preclinical		[93]	
Head and neck cancer				Intratumoral	Phase II	NCT00006106	[94]	Cisplatin, fluorouracil
Melanoma	ZD55-IL-24		Ad2/Ad5, ∆E1B55K, IL-24	Intratumoral	Preclinical		[95]	
Liver cancer				Intravenous	Preclinical		[14]	PEG/lipids/calcium phosphate
Renal cell carcinoma	Ad5-ZD55-CCL5-IL-12		∆E1B55K, CCL5, IL-12	Intratumoral	Preclinical		[22]	CA9-CAR-T
Colorectal cancer	ZD55-CD/5-FU		∆E1B55K, CD/5-FC	Intratumoral	Preclinical		[96]	
Pancreatic cancer	Ad5-yCD/mutTKSR39rep-IL-12		∆E1B55K, HSV-TK. yCD and IL-12	Intratumoral	Phase I	NCT03281382	[97]	5-FC, gemcitabine/paclitaxel
Glioma	BioTTT001	Ad5-TD-nsIL-12	E1∆24, ∆E1B19K, and ∆E3gp19K, IL-12	Intratumoral	Phase I	NCT05717699 NCT05717712		
Pancreatic cancer				Intraperitoneal	Preclinical		[98]	
Colorectal cancer	OAV-CXCL10		∆E1B, CXCL10	Intratumoral	Preclinical		[99]	Anti-PD-1 monotherapy

#### E1B gene modification

The adenovirus E1B locus encodes E1B19K and E1B55K and plays a vital role in the inhibition of apoptosis, inactivation of antioncogene p53 and modulation of viral mRNA export. The viral proteins E1B55K and E4orf6 form an E3 ubiquitin ligase complex that leads to the degradation of p53, which protects infected cells from apoptosis and allows the virus to replicate effectively in normal cells. OAV lacks the ability to bind and inactivate p53 and thus can supposedly replicate efficiently only in neoplastic cells defective in p53 function [100, 101]. ONYX-015 (dl1520), a chimeric adenovirus derived from Ad2/Ad5 with a deleted E1B55K gene, earned the distinction of being the first genetically engineered therapeutic to undergo human trials (Table 3), later gaining commercial approval in China as H101 [13, 102–104]. A more advanced version, ZD55-IL-24, which contains the interleukin (IL-24) and retains a functional E3 region, has shown the ability to effectively combat melanoma by converting tumor cells from the nonself state as well as through the classic direct killing pathway [95].

Despite the promise of the null E1B55K OAV, clinical evaluations suggest that the selectivity mechanism is multifaceted and not solely dependent on p53 status [102, 105–107]. By introducing specific point mutations at the 3' splicing acceptor site of E1B-93R, the novel OAV Ixovex-1 produces the E1B-156R splice isoform and includes a functional E3B region for superior competency compared to OAVs with multiple deletions [108–110]. In models of lung carcinoma, Ixovex-1 significantly enhanced oncolytic efficacy, hindered tumor growth and improved mouse survival [109]. The OAVs associated with E1B modification are listed in Table 3.

#### Tumor-specific promoter driven E1A

To achieve specific replication within tumors, OAV typically uses tumor-specific promoters to regulate E1A gene expression. These promoters are usually inactive in normal cells but are activated in tumor cells, enabling the OAV to replicate only within tumor cells without causing damage to normal cells. The principle behind tumor-specific promoters is the exploitation of the differences in gene expression levels between tumor cells and normal cells to achieve the specific replication of OAV. The replication of OAV controlled by tumor-specific promoter is listed in Table 4. The selective E1A gene and granulocyte macrophage colony-stimulating factor (GM-CSF) encoding CG0070 (Ad5-E2F-E1A-GM-CSF) can replicate and ultimately lyse tumor cells in Rb-deficient tumor cells while releasing tumor antigens and GM-CSF, triggering a systemic antitumor immune response. It has been approved by the FDA for the treatment of nonmuscle invasive bladder cancer (NMIBC). Prior reports of intravesical CG0070 yielded an overall 74.4% complete response (CR) that was maintained for more than 6 months [111, 112]. The Ad3-hTERT-CMV-CD40L leverages human telomerase reverse transcriptase (hTERT) for selective replication, incorporates CD40L as an immune stimulant and avoids the ubiquitous Ad5 NAbs when administered intravenously [113, 114]. Administered intratumorally, OBP-301 (Ad5-hTERT-E1A-IRES-E1B) has displayed cytopathic effects on solid tumors expressing the CXADR-receptor in an hTERT-dependent manner [115, 116]. Given that hypoxia upregulates hTERT activity, compared with Ad5, OBP-301 potentially has enhanced antitumor effects, especially within hypoxic environments [117]. Furthermore, fiber-modified OBP-301 (termed OBP-405) was used to confirm its antitumor effect on non-CXADR-expressing OBP-301-resistant tumors [118].

**Table 4:** OAV replication is controlled by cellular promoters

**Cancer**	**Promoter**	**Vector**	**Construct**	**Transgene/Feature**	**Administration**	**Phase**	**ClinicalTrials. gov ID**	**Ref.**	**Combination**
Bladder cancer	E2F1	CG0070	Ad5-E2F-E1A-GM-CSF	GM-CSF	Intravesical	Phase III	NCT04452591	[111]	N-dodecyl-B-D-maltoside
Melanoma		ICOVIR-5	Ad5-E2F-∆24-RGD4C		Intravenous	Phase I	NCT01864759	[119]	
Solid tumor		ICOVIR-7	Ad5-E2F-palin-∆24-RGD4C	E2F palindromes	Intravenous	Preclinical		[120]	
Solid tumor		ICOVIR-15K-cBiTE	Ad5-E2F-∆24-RGD4C-cBiTE	EGFR-targeting-BiTE	Intratumoral	Preclinical		[121]	PBMC or T cells
Solid tumor		VCN-01	Ad5-E2F-∆24-RGDK-PH20	PH20	Intravenous	Phase I	NCT02045602	[122]	Nab-paclitaxel, gemcitabine
Pancreatic cancer					Intratumoral	Phase I	NCT02045589	[123]	Gemcitabine, abraxane
Solid tumor		VCN-11	Ad5-E2F-∆24-RGDK-PH20-ABD	PH20, albumin-binding domain (ABD)	Intravenous	Preclinical		[124]	
Liver cancer	hTERT	OBP-301	Ad5-hTERT-E1A-IRES-E1B		Intratumoral	Phase I	NCT02293850	[116]	
Esophageal cancer						Phase I	NCT03213054	[125]	Radiotherapy
Solid tumor						Phase I	NCT03172819		Pembrolizumab
Bone and soft tissue sarcomas		OBP-405	Ad5-hTERT-E1A-IRES-E1B-RGD		Intratumoral	Preclinical		[118]	
Pancreatic cancer		OBP-502	Ad5-hTERT-E1A-IRES-E1B-RGD-p53	p53	Intratumoral	Preclinical		[126]	Anti-PD-1 monotherapy
Pancreatic cancer		OBP-702	Ad5-hTERT-E1A-IRES-E1B-RGD-p53	p53	Intratumoral	Preclinical		[127]	Anti-PD-L1 monotherapy
Solid tumor			Ad3-hTERT-E1A			Preclinical		[128]	Chemotherapy
Prostate cancer			Ad3-hTERT-CMV-CD40L	CD40L		Preclinical		[114]	
Solid tumor		KGHV500	Ad5-hTERT-E1A-IRES-E1B-RGD	anti-p21Ras scFv		Preclinical		[129]	
Liver cancer	AFP	SynOV1.1	Ad5-AFP-∆24-RGD-GM-CSF	GM-CSF	Intratumoral	Phase I	NCT04612504		
			Ad5-AFP-NOS- 3/RSV	Nitric Oxide Synthase Type III (NOS-3)	Intravenous	Preclinical		[130]	

### Combination strategies for clinical OAV candidates

Combination strategies may enable the development of efficacious therapies better suited to some specific cancer. Table 1 shows the chimeric OAVs used in clinical practice. For instance, the response to ONCOS-102 (Ad5/3-Δ24-GM-CSF) is associated with increased lymphocyte infiltration and the expression of cytotoxic and costimulatory genes. A phase II study of intratumoral ONCOS-102 revealed substantial immune activation associated with 18-month survival in mesothelioma patients versus chemotherapy alone [64].

Table 2 lists the commercialized OAVs used in clinical trials, each featuring E1A∆24. DNX-2401 (Ad5-Δ24-RGD4C) is modified with E1A∆24 and endowed with the RGD4C motif to its fiber to facilitate entry independent of the CXADR receptor [131]. Intratumoral or intramural infusion of DNX-2401 for glioma intervention has been shown to change T-cell activity and reduce tumor size [74–76]. In a phase II trial of DNX-2401 virotherapy plus pembrolizumab, the overall survival rate was 52.7%, which was significantly greater than the prespecified control rate of 20%, albeit with no statistically significant difference in the objective response rate (ORR) [76]. An enhanced version, DNX-2440, equipped with the costimulatory molecule OX40 ligand (OX40L), can induce tumor-specific immune memory and distant effects [132, 133]. This variant has been or is being investigated in refractory cancers.

As a new favorite, BioTTT001 (Ad5-TD-nsIL-12), which carries triple gene deletions (E1A∆24, ∆E1B19K, and ∆E3gp19K) and nonsecreting (ns) IL-12, can induce tumor cell apoptosis and significantly enhance T-cell infiltration in the TME to inhibit tumor progression [134]. Intratumoral BioTTT001 is also being clinically evaluated against glioma (Table 3). Intraperitoneal delivery of BioTTT001 has been reported to cure peritoneally disseminated pancreatic cancer [98]. The products of this OAV category with E1A 13S or E1B modification are summarized in Table 3.

The OAV whose replication is further controlled by specific cellular promoters is listed in Table 4. A single intravenous administration of ICOVIR-5 (Ad5-E2F-Δ24-RGD4C) can reach tumor sites upon administration but failed to induce tumor regression in a phase I trial for melanoma patients [119]. Similarly, VCN-01 (Ad5-E2F-Δ24-RGDK-PH20) is designed to replicate selectively in tumor cells with a dysfunctional Rb pathway and produces the hyaluronidase PH20 enzyme. This enzyme degrades hyaluronic acid in the tumor extracellular matrix, facilitating drug penetration and immune cell infiltration [123, 135]. In fibrotic tumors such as pancreatic cancer, VCN-01 degrades the tumor stroma and remodels the TME, representing a new therapeutic agent for cancers with dense stroma [44, 123]. Clinical trials have shown that its combination with chemotherapy facilitates the delivery of a variety of therapeutic agents and has a 50% ORR in pancreatic cancer patients [136]. VCN-11, an evolved version of VCN-01, not only expresses the hyaluronidase PH20 but also features an albumin-binding domain (ABD) on hexon, allowing the virus to evade NAbs in the bloodstream. Preliminary studies have shown minimal side effects and enhanced tumor targeting via intravenous route [124].

## rAd-based immunostimulatory therapy

### Enhancing immunotherapy with rAd-induced immunostimulation

**Fig. 3:**
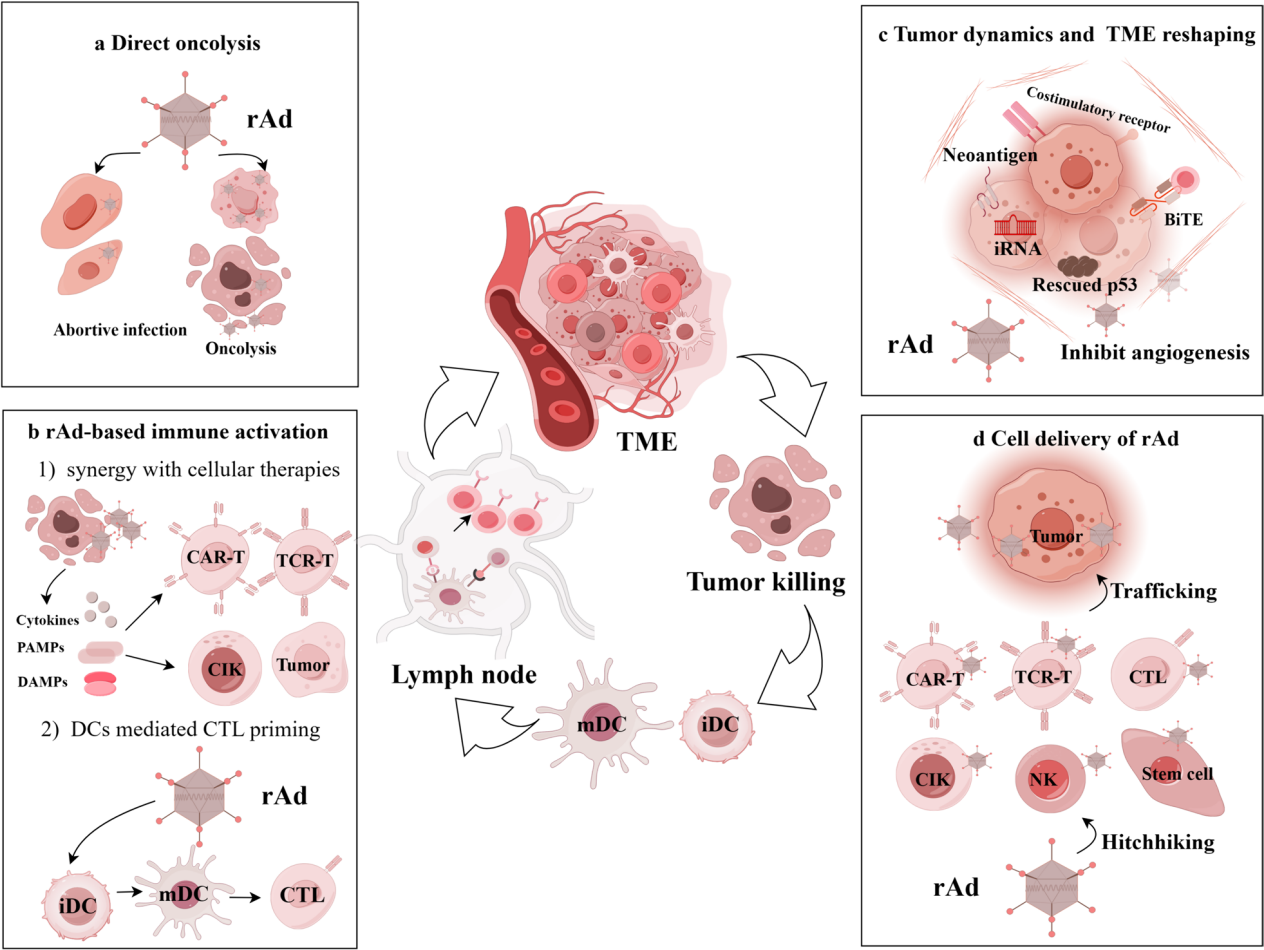
Application framework of rAd in cancer immunotherapy. **a** Direct oncolysis **b** rAd-based immune activation: DC vaccine mediated CTL priming and rAd synergy with cellular therapies **c** Reshaping tumor dynamics and 'firing up' the TME **d** Cell delivery of rAd to tumor sites. The evoked immune cells activate TME and induce the infiltration of more immune cells, thereby enhancing local and systemic anti-tumor immunity

Immunostimulatory genes play a pivotal role in orchestrating immune responses against cancer. The strategy of using rAd to transduce immunostimulatory genes such as TAAs, costimulatory molecules [6, 137, 138], and cytokines such as (C-C motif) ligand 5 (CCL5), IL, interferon (IFN), GM-CSF, and tumor necrosis factor (TNF) [12, 139–144] has shown significant promise in enhancing tumor immunogenicity and 'firing up' the TME (Fig. 3). Additionally, immune checkpoint inhibitors (ICIs) against SOCS1, CTLA4, PD1, TIGIT, etc., have been incorporated into rAd for cancer immunotherapy [145, 146].

rAd is increasingly recognized as an effective virotherapy in clinical settings, offering both systemic and local therapeutic approaches (Table 5) [12, 147, 148]. For instance, in a phase I clinical study, subcutaneous administration of the multitargeted vaccine Ad5-PSA/MUC1/Brachyury or Ad5-CEA/MUC1/Brachyury induced immunogenicity and was well-tolerated, with a median progression-free survival of 22 weeks [149, 150]. All patients mounted T-cell responses to at least one TAA, whereas 47% of patients mounted immune responses to all three TAAs [149]. In a phase III clinical trial, intravesical injection of Ad5-IFNα/Syn3 expressing IFNα and Syn3 (a polyamide surfactant used to enhance viral transduction) demonstrated efficacy in treating NMIBC. Fifty-five (53.4%) of the 103 patients achieved a complete response within 3 months, and this response was maintained in 25 (45.5%) of the 55 patients at 12 months [151].

**Table 5:** Therapeutic genes transferred by rAd in clinical trials

**Cancer**	**Construct**	**Transgene/Feature**	**Administration**	**Phase**	**ClinicalTrials.** **gov ID**	**Ref.**	**Combination**
Prostate cancer	Ad5-PSA/MUC1/Brachyury	Prostate-specific antigen (PSA), mucin-1 (MUC1), brachyury	Subcutaneous	Phase I	NCT03481816	[149]	Radiation therapy
Solid tumors	Ad5-CEA/MUC1/Brachyury	Carcinoembryonic antigen (CEA), MUC1, brachyury	Subcutaneous	Phase I	NCT03384316	[150]	
Bladder cancer	Ad5-IFNα/Syn3 (Nadofaragene firadenovec)	IFNα2b, syn3	Intravesical Intravesical Intravesical	Phase III Phase II Phase I	NCT02773849 NCT01687244	[151] [152] [153]	
Breast cancer	Ad5CMV-p53	p53	Intratumoral	Phase I	NCT00004038		Chemotherapy
			Intratumoral	Phase II	NCT00044993	[154]	Chemotherapy
Oral or pharyngeal cancer			Intratumoral	Phase II	NCT00064103		
Solid tumor			Intratumoral	Phase II	NCT03544723		Immune checkpoint inhibitors
Lung cancer	DC-rAd.p53	p53, DC vaccine	Intradermal	Phase II	NCT00617409	[155]	Paclitaxel, all -trans retinoic acid
Breast cancer			Intradermal	Phase II	NCT01042535	[156]	Indoximod
Acute myeloid leukemia (AML)	rAd-siSSF	Survivin, MUC1, shRNA DC vaccine	Inguinal	Phase I	NCT01956630	[145]	Allogenic hematopoietic stem cell transplantation
Head and neck cancer	Ad5-PNP	purine nucleoside phosphorylase (PNP)	Intratumoral	Phase I	NCT01310179	[157]	F-araAMP
Glioma	Ad5-TK, rAd-Flt3L	Thymidine kinase (TK), Fms-like thyrosine kinase 3 ligand (Flt3L)	Intracranial	Phase I	NCT01811992	[158]	
Breast cancer	Ad5-TK		Intratumoral	Phase II		[159]	Pembrolizumab
Glioma	Ad5-CD/TKrep	TK, cytosine deaminase (CD)	Perilesional	Phase III	EudraCT, number 2004-000464-28	[160]	Ganciclovir
Prostate cancer			Intraprostatic	Phase I	NCT00583492	[161]	Ganciclovir, 5-FU
			Intraprostatic	Phase I	NCT00583492	[162]	Radiation therapy, anciclovir, 5-FU

### rAd-activated DCs as vaccines

DCs orchestrate adaptive immunity by taking up and presenting antigens to T cells. Due to the potent ability of DCs to prime naive T cells, there has been longstanding interest in DC vaccines, particularly when activated by rAd infection (Fig. 3b). Notably, rAd not only efficiently promotes DC maturation and robust immunogenicity but also has a natural affinity for DCs *in vitro* [163, 164]. Despite lacking viral CXADR, DCs can internalize Ad5 through phagocytosis [165]. However, direct intravenous administration of rAd reduces the endocytic and cross-presentation capabilities of DCs, compromising the priming of CTLs [166].

#### rAd-mediated tumor antigen expression in DCs

Protective immunity can be generated with TAA-engineered DC vaccines [167, 168]. Unlike peptide-pulsed DCs, rAd-transduced DCs induce DC differentiation and maturation, expanding the range of HLA applications. rAd-induced DC vaccines can process entire TAAs into peptide pools, enabling recognition by the corresponding T-cell receptors (TCRs) and the generation of specific CTLs [169]. Furthermore, the CTL priming with viral or virally encoded epitopes gives rise to enhanced proliferation, infiltration, and distinct memory phenotypes. Preclinical studies have demonstrated the superior efficacy of DC vaccines containing rAd-encoded tyrosinase-related protein 2 (TRP2) compared to direct rAd-TRP2 injections, thus highlighting the robust immunogenicity of rAd-induced DCs [170].

#### rAd-induced costimulatory molecules and cytokines in DCs

To overcome immune tolerance, rAd has been engineered to deliver immunoregulatory genes and cytokines into DCs. This strategy enhances Th1 and CTL responses, which are crucial for effective antitumor immunity [171–175]. For instance, rAd-IL-18 transgene-engineered DC vaccines have shown potential due to the unique ability of IL-18 to induce tumor-specific CTLs [176, 177]. Similarly, compared with their externally cultured counterparts, rAd-IL-6-engineered DC vaccines enhance specific CTL responses and counter immunosuppression [178], while rAd-TNFα-transfected DCs exhibit greater maturation and T-cell activation [179]. The use of the rAd-CD137L vaccine promoted DC-induced priming of tumor-specific CD8^+^ T cells [7]. Additionally, Ad5F35-CD40L-IL-2 vaccines contribute to DC maturation and IL-12 production by targeting breast cancer cells overexpressing CD40 [180]. However, immature DCs transduced with rAd-CD40L can differentiate into tolerogenic DCs [181]. The complex interplay between costimulatory molecules and cytokines in DC maturation and CTL functionality has yet to be elucidated.

By collecting the lysate of tumor cells infected with replication-competent Ad3-hTERT-CMV-CD40L and culturing them with DCs, DC maturation and the production of proinflammatory cytokines can be induced, thereby augmenting the effectiveness of DC vaccination [114, 182]. Additionally, a nanovaccine derived from rAd-infected mature DCs in which specific MHC-I, anti-PD1 antibody and B7 costimulatory molecules are simultaneously anchored can self-present neoantigens to T cells and stimulate strong CTL responses in this manner [183].

#### Clinical trials of rAd-based DC vaccines

INGN-225 is an rAd-mediated p53-expressing DC vaccine (DC-rAd.p53). In phase II clinical trials of recurrent small cell lung cancer (Table 5), INGN-225 was demonstrated to induce significant immune responses and improve the efficacy of chemotherapy but failed to improve the ORR [155, 156, 184]. Moreover, in addition to flagellin, rAd delivers survivin and MUC1 TAAs to promote DC maturation, and a shRNA is used to suppress SOCS1, an intracellular immune checkpoint molecule (rAd-siSSF). Via inguinal injection, a phase I trial of rAd-siSSF demonstrated its safety and efficacy, with a complete remission rate of 83% in relapsed acute myeloid leukemia (AML) patients [145].

### rAd as immunostimulant in cellular therapies

While rAd can effectively deliver therapeutic payloads to tumors, transient expression of these viruses necessitates repeated high-titer injections to maintain effective local concentrations [185, 186]. This strategy, combined with other therapies, is anticipated to significantly boost antitumor immunity (Fig. 3c) [142, 187, 188].

#### CAR-T cell and rAd synergy

Intratumorally administering rAd combined with CAR-T cells can overcome the challenges of the immunosuppressive TME and promote CAR-T cell infiltration and proliferation *in vivo* to eradicate local and distal tumors. Cytokine-armed OAV expressing TNFα and IL-2 (Ad5/3-E2F-Δ24-TNFa-IRES-IL-2), in conjunction with mesothelin-targeted CAR-T cells, foster T-cell infiltration and promote M1 macrophage polarization and DC maturation [189]. Moreover, the replication competent Ad5-ZD55-CCL5-IL-12, which encodes the chemokines CCL5 and IL-12, significantly increases CAR-T-cell infiltration in tumors, extending survival and restraining tumor growth. Since IL-12 enhances the phosphorylation of signal transducer and activator of transcription 4 (STAT4) in CAR-T cells and stimulates IFNγ release [22], local treatment with rAd-IL-12-PD-L1, which encodes a PD-L1-blocking antibody, and IL-12p70 was found to control both primary and metastasized head and neck squamous cell carcinoma in conjunction with HER2-specific CAR-T cells [190].

#### Other immunocyte therapies augmented by rAd

The rAd has documented to augment the antitumor effects of CIKs for cancer immunotherapy [191, 192]. The novel hydrogel-mediated codelivery of replication-competent rAd-IL-12-IL-15 and CIK cells could enhance the combined antitumor effects. This strategy involving an injectable and biodegradable hydrogel minimizes the dispersion of high-dose OAV and CIK cells, thus reducing nontumor exposure [193]. Another example is the intratumoral injection of ICOVIR-15K-BiTE, which expresses a bispecific T-cell adapter (BiTE) simultaneously with T-cell infusion; this method can bidirectionally recognize neoplastic cells and T cells and induce T-cell activation and tumor cell destruction [121].

#### rAd in tumor cell vaccination

The use of autologous unirradiated tumor cells transduced with rAd-IL-12 has shown promise in treating advanced neuroblastoma. This approach induces both a local inflammatory response and a systemic immune response, characterized by an increase in the number of circulating CD25^+^ and DR^+^CD3^+^ T cells and specific antitumor responses [194]. Likewise, cryoshocked tumor cells, which constitute an OAV reservoir, can eliminate viral proliferation and pathogenicity, steadily release viruses and efficiently initiate an endogenous antitumor response by increasing memory T cells and modulating the TME [195, 196]. More recently, extracellular vesicles derived from OAV-infected tumor cells have been used for delivering immunogenic OAV, inducing systemic immune responses through proinflammatory cytokines, and inhibiting primary and metastatic cancers [77, 78].

## rAd reshaping tumor dynamics and the TME

### rAd delivery of RNA interference (RNAi) against oncogenes

rAd has been demonstrated to enhance anti-tumor effects by expressing RNAi, targeting oncogenes or immunosuppressive genes such as PD1 and K-RAS (Fig. 3c). Notably, STAT3 is considered as a bona fide oncogene and mediates immunosuppressive functions in various immune cells including macrophages, myeloid-derived suppressor cells, and DCs [197, 198]. Given the challenges in inhibiting STAT3 through antibodies or small molecule inhibitors, siRNA serves as an ideal alternative for STAT3 inhibition. Depleting STAT3 in DCs improves their antigen-presenting activity and enhances antitumor immune responses [199]. Additionally, inhibiting STAT3 in cancer cells promotes ICD and increases IFN-responsive chemokines, facilitating immune cell infiltration [200].

### rAd-rescued tumor suppressor gene expression

Tumor suppressor genes, which are crucial for inhibiting cell proliferation and tumorigenesis, often undergo mutation or inactivation in malignant tissues. rAd can target gene defects in key tumor suppressor genes, such as p53, p16/21/27, Rb, and PTEN [201–203]. For example, restoration of p53 in p53-deficient tumor cells has been shown to suppress tumor growth or induce apoptosis in both *in vitro* and *in vivo* models. Ad5CMV-p53, an oral infusion or intramucosal injection, has the potential to prevent oral or pharyngeal precancerous lesions, with an estimated 1-year progression-free survival rate of 92% (Table 5). In the case of the phase II trial of Ad5CMV-p53 combined with chemotherapy, the estimated 3-year survival rate was 84% [154].

### rAd-mediated "suicide gene" for toxic molecule delivery

rAd can also target tumor cells by expressing enzymes that convert prodrugs into toxic compounds (Table 5). For instance, purine nucleoside phosphorylase (PNP) converts fludarabine phosphate (F-araAMP) into cytotoxic fluoroadenine. The first-in-human clinical trial found that rAd-PNP combined with intravenous F-araAMP shows potential in treating advanced glioma [204]. With herpes simplex virus thymidine kinase (HSV-TK) transforming ganciclovir into nucleotides toxic to dividing cells, rAd-TK plus ganciclovir therapy has been employed in numerous clinical trials for solid tumor treatment [158]. Additionally, cytosine deaminase (CD) converts the prodrug 5-fluorocytosine (5-FC) into toxic 5-fluorouridine (5-FU), which is metabolized into 5-fluorouracil triphosphate (5-FUTP) and 5-fluorodeoxyuridine monophosphate (5-FdUMP), causing thymidylate synthesis blockade and DNA damage. Concomitant with prodrug therapy of 5-FC and ganciclovir, clinical trials showed that replication-competent Ad5-CD/TKrep carrying CD and TK chimeric genes exerted long-term effectiveness [161, 162].

### rAd in reshaping the TME

To inhibit tumor angiogenesis, vasohibin has been identified as an intrinsic and specific angiogenesis inhibitor. The therapeutic potential of the rAd-vasohibin, which encodes vasohibin, has been explored. When administered via tail vein injection, rAd-vasohibin prevented tumor cell growth in a subcutaneous tumor model by inhibiting angiogenesis without apparent side effects [205]. Additionally, targeting cancer-associated fibroblasts (CAFs) in the immunosuppressive TME is crucial. Fibroblast activation protein (FAP), a cell surface serine protease highly expressed on CAFs, was targeted by rAd-FAP. As a result, rAd-FAP enables *in vivo* gene delivery to stromal cells in the TME, resulting in attenuated tumor growth [206].

## Synergistic interaction between rAd and cellular therapeutics for targeting tumor sites

The ability of rAd to synergistically interact with various cell types, particularly immune cells, creates a potent combination for targeting tumor sites and potentiates cellular therapies in oncology (Fig. 3d). The advantage of this approach lies in its dual functionality: while rAd can enhance the tumor-targeting ability and therapeutic payload delivery of cells, as mentioned before, the cells themselves offer a biologically compatible and dynamic method for rAd transport. This is particularly relevant in residual and metastatic tumors, where the physical barriers and immunosuppressive nature of the TME pose significant challenges for conventional therapies.

### rAd hitchhiking on/in immune cells

For oncolytic virus (OV) delivery, CAR-T and TCR-T cells have been proven to be effective vehicle cells, especially for oncolytic vesicular stomatitis virus (VSV), myxoma virus (MYXV) and reovirus [23, 25]. In previous studies, OV-loaded CAR-T cells were shown to have enhanced activity and efficacy against solid tumor models and to augment expansion rate *in vivo* via homologous boosting [25]. OVs loaded in/on cells can be directly delivered into solid tumors in a CAR/TCR-directed fashion, avoiding recognition by the host innate defense system [23]. Analogously, Epstein-Barr virus (EBV)-specific CTLs provide an innovative approach for delivering therapeutic Ad5F35 to tumor sites, not only to locally accessible macroscopic tumors, but also to disseminated metastatic disease [24]. These CTLs, which are transgenic for the adenoviral E1 gene under the CD40L promoter, produce and release infectious Ad5F35 upon exposure to HLA-matched EBV-expressing targets but not in response to HLA-mismatched or irrelevant cells, which can avoid the risks associated with systemic administration of large doses of rAd.

Replication-competent KGHV500, carrying anti-p21Ras single-chain variable fragment antibody (scFv), has shown potential for blocking the Ras signaling pathway and inhibiting Ras-driven cancers. In several Ras-driven cancers, the CIK cell-based delivery of KGHV500 has been validated through both *in vitro* and *in vivo* studies, which confirmed the tumor-targeting efficacy and systemic safety of OAV-loaded CIK cells [129, 207–209]. Likewise, the oncolytic agent ZD55 introduces the CD40L promoter to regulate replication, ensuring that cell proliferation is strictly controlled by CIK cell activation. This targeted delivery by CIK cells enhances antitumor efficacy and precision in tumor targeting and minimizes infection in nontumor tissues [210].

With their innate tumor-homing ability, NK cells function as bioreactors that support OAV loading, protection, replication, amplification, and targeted release. Arming NK cells with OAV not only boosts antitumor immunity through IFN signaling but also alleviates immunosuppression in the TME, promoting DC maturation and M1 macrophage polarization. Both *in vitro* and *in vivo* data highlight the potent antitumor and antimetastatic functions of this NK cell-mediated OAV delivery system [211].

### Use of stem cell as carriers of rAd

The abilities to home to tumors, shield rAd from host antiviral responses, and infiltrate tumor tissues through the TME make stem cells ideal candidates as delivery vehicles for rAd in cancer therapy [28, 212–215]. As listed in Table 6, mesenchymal stem cells (MSCs) transduced with the IFNβ expressing nonreplicating rAd (MSC-rAd.IFNβ) have shown promise in suppressing pulmonary metastasis through IFNβ production within the TME [216]. MSCs efficiently delivered rAd expressing IL-12 (MSC-rAd.IL-12) in glioma but does not completely arrest the invasive growth pattern of these lesions [217]. However, MSCs transduced with rAd carrying the secretable trimeric form of tumor necrosis factor-related apoptosis-inducing ligand (TRAIL) (MSC-rAd.stTRAIL) significantly inhibited tumor growth and prolonged survival in glioma-bearing mice [48, 218].

**Table 6:** Clinical and preclinical trials using stem cells as delivery vehicle for OAV

**Cancer**	**Vehicle**	**Construct**	**Transgene/Feature**	**Administration**	**Phase**	**ClinicalTrials. gov ID**	**Ref.**	**Combination**
Liver cancer	MSC	MSC-rAdCD3scFv	anti-CD3scFv		Preclinical		[219]	LentiR.E1A, 5-FU
Solid tumor		MSC-rAd.IFNβ	IFNβ	Intravenous	Preclinical		[216]	
Glioma		MSC-rAd.IL-12	IL-12	Peritumoral	Preclinical		[217]	
Glioma		MSC-rAd.stTRAIL	PTD, stTRAIL	Intratumoral	Preclinical		[218]	
Ovarian cancer		MSC-Ad5pK7-meso64-TR3	Truncated mesothelin, TRAIL, pk7	Intraperitoneal	Preclinical		[47]	
Ovarian cancer Glioma		MSC-Δ24RGD		Intravenous	Preclinical		[213, 220]	
Glioma		MSC-Ad5F35-Tet-on-E1BPro-∆24-IL-24/endostatin	IL-24/endostatin	Intravenous	Preclinical		[221]	
Pancreatic cancer		MSC-Ad5F3-∆E1B19K-TRAIL	∆E1B19K, TRAIL		Preclinical		[222]	
Colorectal cancer		MSC-Ad5F11-hTERT-E1AΔ24		Intraperitoneal	Preclinical		[223]	
Liver cancer		MSC-AdAFPp-E1A-miR122	AFP promoter,microRNA-122	Intratumoral	Preclinical		[224]	
Liver cancer		MSC-rAd-E1A-αCD3HAC	BiTE targeting the PD-L1 and CD3	Intravenous	Preclinical		[225]	PBMCs
Liver cancer		MSC-Ad5F35-Ha2bm-E1A-WNTi	Ha2bm promoter, WNTi	Intravenous	Preclinical		[26]	
Glioma	NSC-CRAd-S-pK7	NSC-CRAd-Survivin-pK7	pK7	Intracranial	Phase I	NCT03072134 NCT05139056	[30]	Tumor resection, temozolomide and radiotherapy Tumor resection
Glioma	MSC-DNX-2401	MSC-Ad5-∆24-RGD4C		Intraarterial	Phase I	NCT03896568	[226]	Tumor resection
Glioma	MSC-ICOVIR-17	MSC-Ad5-E2F-∆24-RGD-PH20	PH20	Intratumoral	Preclinical		[227]	
Melanoma	MSC-ICOVIR-5	MSC-Ad5-E2F-∆24-RGD		Intravenous	Phase I	NCT01864759	[228]	Chemotherapy and radiotherapy
Solid tumor				Intravenous	Phase II	NCT01844661		
Glioma				Intravenous	Phase II	NCT04758533		

In terms of replication-competent rAd, the intraperitoneal administration of MSC-preloaded OAVΔ24RGD (MSC-Δ24RGD) can efficiently target ovarian tumor cells and reduce the systemic toxicity of naked virions in mice [220]. In a glioma xenograft murine model, MSC-guided delivery of MSC-Δ24RGD could migrate to tumors and exert antitumor effects via intravenous injection [213]. Furthermore, the endovascular selective intra-arterial (ESIA) infusion of MSC-Δ24RGD is a rapidly evolving strategy for treating glioma in a clinically relevant fashion [229]. In seeking to increase the viral infection and production by MSCs, a bunch of OAV chimerisms have been designed [223]. Engineered Ad5F3 expressing the TRAIL or FCU1 would enhance oncolysis by improved virus production in MSCs, thereby implementing delivery into established and primary pancreatic cancer cells [222]. In a xenograft model of glioma, systemic administration of MCSs loaded with Ad5F35 carrying IL-24 and/or endostatin and regulated by a Tet-on system (Ad5F35-Tet-on-E1B-Pro-Δ24-IL-24/endostatin) showed promise for glioma treatment while sparing normal cells [221].

In the context of hepatocellular carcinoma (HCC), MSCs loaded with OAV have demonstrated the ability to home to HCC cells and differentiate into hepatocyte-like cells within the TME [224]. They effectively package and release progeny virions that contain an adenovirus E1A gene regulated by the α-fetoprotein (AFP) promoter and microRNA-122 target sequence (AdAFPp-E1A-miR122), resulting in dramatic tumor inhibition in mouse models. Another oncolytic Ad5F35, which can replicate under the control of the AFP-positive HCC-specific Ha2bm promoter and express the WNT inhibitory (WNTi) bait receptor, was transfected into MSCs, which were then intravenously injected into HCC-bearing mice. Compared with WNTi without a cell carrier, the therapeutic effect was much more satisfactory [26]. Newer versions of OAV armed with BiTE have shown enhanced antitumor effects, reduced liver injury, and improved T-cell infiltration and activation in orthotopic transplantation model mice [225]. In conclusion, the use of rAd-mediated hitchhiking of MSCs has demonstrated safety in preclinical studies and a pleiotropic profile of tumor destruction.

### Clinical trials of rAd hitchhiking

A pioneering trial of children with relapsed or refractory neuroblastoma used autologous MSCs carrying ICOVIR-5 (Ad5-E2F-Δ24-RGD4C; Table 6). The trial reported disease stabilization or remission in patients, with no toxicity or progressive disease observed [228]. This groundbreaking study paved the way for further exploration of the use of ICOVIR-5-loaded MSCs in treating late-stage solid tumors in both adults and children. Two additional ongoing trials (NCT01864759 and NCT04758533) are utilizing ICOVIR-5 in conjunction with allogeneic MSCs, to treat advanced or metastatic melanoma and gliomas. However, as of the last update, the results from these trials have not been reported. Concordantly, a separate study involving MSCs loaded with hyaluronidase-expressing ICOVIR-17 demonstrated a significant reduction in glioblastoma growth and increased survival in a clinically relevant murine model [227]. This approach is being further investigated in a phase I clinical trial (Table 6), where autologous MSCs loaded with DNX-2401 are evaluated for their therapeutic effects on glioblastoma with the support of endovascular super-selective intra-arterial infusion [226].

The commercial OAV loaded in neural stem cells has been underway [132, 230]. The safety and efficacy of the neural stem cell delivered CRAd-Survivin-pK7 (NSC-CRAd-Survivin-pK7) under the survivin promoter were evaluated in the first human clinical trial of malignant glioma, in which the median progression-free survival was 9.1 months and the median overall survival was 18.4 months (Table 6) [30].

## Conclusions and future perspectives

In this review, we present an integral toolkit for rAd application according to four different antitumor mechanisms (Fig 3), i.e., a) direct oncolysis, b) immune activation: DCs priming CTL; synergies with cellular therapies, c) reshaping tumor dynamics and 'firing up' the TME, d) tumor targeting by carrier cells. With their natural tropism for epithelial cells, adenoviruses are inherently suited for targeting the majority of solid tumors. The strategic utilization of rAd in cancer therapy, particularly through replication-competent OAV, has shown significant promise. Despite challenges such as immune reactions against the virus, ongoing clinical evaluations of various rAd-equipped ACTs and combined cancer immunotherapy strategies are yielding encouraging results in terms of both safety and efficacy. In addition, the integration of immune cells and/or stem cells with rAd is pivotal in understanding cell-type-specific oncolysis within the TME and controlling tumor growth through diverse mechanisms. The development of effective rAd delivery vehicles is crucial, with criteria including high cellular infection rates, effective gene expression or replication, and the capability of carrier cells to target tumors.

Of particular interest is the role of rAd in CAR-M therapy. Macrophages, critical players in tumor progression, can be genetically modified ex vivo for adoptive transfer [231]. CAR-M, representing a groundbreaking approach in immunotherapy, exhibits functions like targeted phagocytosis, induction of a proinflammatory M1 phenotype, antigen presentation, and epitope spreading. Early studies have shown the effectiveness of CAR-M against blood tumors and solid tumors, like ovarian cancer, highlighting its potential to reshape TME [232, 233]. However, the application of CAR-M faces challenges. Macrophages' inherent defense mechanisms against viral infections, such as the production of antiviral IFNs and undergoing apoptosis, make them less susceptible to common viral vectors used in gene delivery. This challenge is addressed by the discovery of Ad5F35 as an efficient gene carrier for macrophages, leveraging their abundant CD46 expression. Ad5F35-infected macrophages not only demonstrate antigen-specific phagocytosis but also maintain a sustained proinflammatory M1 phenotype and present tumor antigens to T cells [234–236]. Importantly, CAR-Ms, especially those transduced with Ad5F35, have shown potential in priming T cells against neoantigens, reducing antigen escape and recurrence.

In summary, engineering rAd holds immense potential for enhancing cellular immunity and synergistically improving anti-tumor effects alongside other novel cancer immunotherapies. As research progresses, the unique advantages of rAd in targeting and reshaping the TME combined with innovative approaches will become increasingly significant in anti-tumor immunity.

## Data Availability

No datasets were generated or analysed during the current study.

## Abbreviations

5-FC: 5-fluorocytosine
5-FdUMP: 5-fluorodeoxyuridine monophosphate
5-FUTP: 5-fluorouracil triphosphate
5-FU: 5-fluorouridine
E1A∆24: A 24bp constant deletion in the E1A CR2
∆24: A 24bp constant region deletion
scTCR: A single-chain T-cell receptor
F/K20: A stretch of 20 lysine residues
AML: Acute myeloid leukemia
Ad5: Adenovirus type 5
ACT: Adoptive cell therapy
ABD: Albumin-binding domain
αv: Alpha V
RGD: Arg-Gly-Asp motif
β3: Beta 3
β5: Beta 5
BiTE: Bispecific T cell engager
CAFs: Cancer-associated fibroblasts
CCL5: Chemokine (C-C motif) ligand 5
CAR-M: Chimeric antigen receptor macrophages
CAR-T: Chimeric antigen receptor T cells
CR: Complete response
CRAd: Conditionally replicating adenovirus
CR: Conserved region
CXADR: Coxsackievirus-adenovirus receptor
CIK: Cytokine-induced killer cells
CD: Cytosine deaminase
CTL: Cytotoxic T lymphocytes
DAMPs: Damage-associated molecular patterns
DC: Dendritic cells
DSG2: Desmoglein 2
E1-E4: Early genes
ESIA: Endovascular selective intra-arterial
EBV: Epstein-Barr virus
FAP: Fibroblast activation protein
F-araAMP: Fludarabine phosphate
GM-CSF: Granulocyte macrophage colony-stimulating factor
HSV-TK: Herpes simplex virus thymidine kinase
HLA: Human leukocyte antigen
hTERT: Human telomerase reverse transcriptase
ICI: Immune checkpoint inhibitors
ICD: Immunogenic cell death
IFN: Interferon
IL-24: Interleukin
iRGD: Internalizing RGD
ITR: Inverted terminal repeats
L1-L5: Late genes
LOAd: Lokon oncolytic adenovirus
MAGE: Melanoma-associated cancer-testis antigen
MSC: Mesenchymal stem cell
MYXV: Myxoma virus
NK: Natural killer cells
NAbs: Neutralizing antibodies
NMIBC: Nonmuscle invasive bladder cancer
ns: Non-secreting
ORR: Objective response rate
OAV: Oncolytic adenovirus
OVs: Oncolytic viruses
OX40L: OX40 ligand
PAMPs: Pathogen-associated molecular patterns
pK7: Polylysine motif
PNP: Purine nucleoside phosphorylase
rAd: Recombinant adenovirus
Rb: Retinoblastoma protein
RNAi: RNA interference
STAT4: Signal transducer and activator of transcription 4
scFv: Single-chain variable fragment antibody
TCR-T: T cell receptor-engineered T cells
TCRs: T cell receptors
TME: Tumor microenvironment
TNF: Tumor necrosis factor
TRAIL: Tumor necrosis factor-related apoptosis-inducing ligand
TAAs: Tumor-associated antigens
TIL: Tumor-infiltrating lymphocytes
TSAs: Tumor-specific antigens
TRP2: Tyrosinase-related protein 2
VSV: Vesicular stomatitis virus
WNTi: WNT inhibitory
AFP: Α-fetoprotein
